# Retrospective Analysis of Intravaginal Brachytherapy in Adjuvant Treatment of Early Endometrial Cancer

**DOI:** 10.1155/2018/7924153

**Published:** 2018-02-21

**Authors:** Paweł Cisek, Dariusz Kieszko, Izabela Kordzińska-Cisek, Elżbieta Kutarska, Ludmiła Grzybowska-Szatkowska

**Affiliations:** ^1^Department of Oncology, Medical University of Lublin, Jaczewskiego 7, Lublin, Poland; ^2^Department of Brachytherapy, St. John's Oncology Center of Lublin, Jaczewskiego 7, Lublin, Poland; ^3^Department of Gynecology, St. John's Oncology Center of Lublin, Jaczewskiego 7, Lublin, Poland

## Abstract

The aim of this study was to determine the role of adjuvant endovaginal brachytherapy HDR (High Dose Rate) or observation, as well as identification of risk factors of tumor recurrence. The study included 178 women after radical hysterectomy. All patients belonged to the group of low- and medium-risk stage I FIGO. Analysis consisted of 3-, 5-, and 10-year OS, DFS, and LRFS in both groups. Follow-up was more than 6.5 years. The 5-OS, 5-DFS, and 5-LRFS were 93%, 96%, and 98% in the treated group and 95%, 94%, and 96% in the observed group, respectively. These differences were not statistically significant. There was a statistically significant difference in 5-OS in the treated group, between low- and medium-risk subgroups (100% versus 87.55%, *p* = 0.018). There was a better prognosis among the patients with FIGO IA compared to FIGO IB (5-DFS, 97 versus 86%, *p* = 0.047). Among the risk factors, there were only statistically significant differences in the 5-OS, between the ages of ≤ 70 years and >70 years. Use of brachytherapy may affect the reduction in the number of local recurrences at the vaginal stump (6% versus 2%). This is particularly noticeable in the low-risk subgroup (9% versus 0%).

## 1. Introduction

Uterine cancer is the sixth most common cancer in women in the world and the fourth most common cancer in Europe. It is also the second most common cancer among gynecological cancers after cervical cancer in the world and first in Europe [1]. The management of endometrial cancer is complex and it is based on surgical treatment, radiotherapy, chemotherapy, and hormonal therapy. A recommended and routine surgical procedure is the total abdominal hysterectomy with bilateral salpingooophorectomy [2]. The role of pelvic lymphadenectomy has not been definitively established and remains controversial [3]. Depending on the stage of cancer, exclusive observation, teleradiotherapy, brachytherapy, a combination therapy involving both of these methods, or chemotherapy are used after surgery [2].

In general, no supplementary treatment [2] is used in the low-risk group (IA, G1, G2, and endometrioid type). Literature analysis indicates no benefit in overall survival from complementary radiotherapy [4–8]. There are also no new, large randomized trials comparing complementary brachytherapy with exclusive observation in this group of patients. The decision regarding complementary treatment or follow-up in other patients with FIGO (International Federation of Gynecology and Obstetrics) IA is usually based on the presence of a number of factors, which are considered unfavorable prognosis. These include the following: age > 70 years, histopathological type II or mixed (according to Bokhman), low tumor descent, tumor mass > 2 cm, low uterine segment involvement (LUSI), and histopathological grade G3 [2]. There is even more controversy about patients with FIGO IB—there are no studies comparing exclusive surveillance with independent brachytherapy in this group of patients. Treatment usually depends on the presence of risk factors, the hospital's own experience, and the patient's preference. In this study, we performed a one-sided, comparative retrospective analysis of patients with early-stage endometrial cancer treated with complementary brachytherapy or postoperative follow-up.

## 2. Material and Methods

### 2.1. Characteristics of Patients

**Figure 1:**
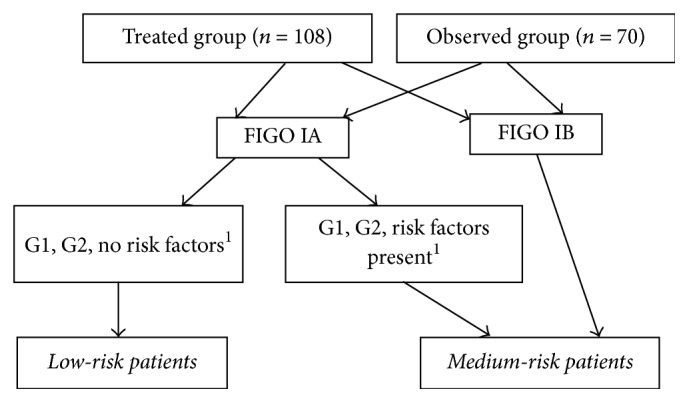
Division into prognosis subgroups. ^1^Risk factors of recurrence: histopathological type II or mixed, low tumor descent, tumor size > 2 cm, age > 70 years, and degree of malignancy G3.

The retrospective analysis included 178 patients with histopathologically confirmed invasive endometrial cancer in the first stage of FIGO clinical stage, treated by brachytherapy or subjected to a control study in 1989–2013. All patients were after total abdominal hysterectomy with bilateral salpingooophorectomy. Node dissection was optional. All patients were treated with a radical premise. The patients were divided according to the scheme shown in Figure 1 into two groups:

1. Treated group: who received adjuvant brachytherapy
2. Observed group: who used only observation.

Then, depending on clinical and histopathological factors, the two groups were divided according to the scheme shown in Figure 1 into patients with low and medium risk.

There were 49 low-risk and 59 medium-risk patients in the treated group and 32 low-risk patients and 38 medium-risk patients in the observed group (Table 1).

### 2.2. Treatment

Patients from both groups had undergone uterine abduction with pelvic lymphadenectomy or no lymphadenectomy. Patients in the treated group were irradiated by HDR (High Dose Rate) brachytherapy using Ir192 source. The treatment was started from the placement of a single-tube cylindrical applicator into the vagina and an X-ray examination to plan the treatment. Treatment planning was to determine the source stops and stopping time of the source in the applicator to cover with the prescribed isodose the upper 1/3 part of the vaginal mucosa at a depth of 5 mm. The dose was also calculated at the ICRU (International Commission on Radiation Units and Measurements) of the bladder and rectum and the maximum dose in the vaginal mucosa. Median fractional dose was 7.5 (6–8) Gy and total dose 30 (15–32) Gy. Most 3-4 fractions were used at weekly intervals.

Patients undergoing exclusive follow-up and postbrachytherapy patients were assigned for a follow-up visit every three months during the first two years and then every six months and every five years. The primary outcome measure was the 3-, 5-, and 10-year OS, DFS, and LRFS rates, depending on the type of follow-up and prognosis. The second outcome was identification of risk factors of tumor recurrence. Statistical analysis was performed with the Kaplan-Meier method along with the log-rank test.

## 3. Results

### 3.1. Overall Survival (OS)

Patients included in the study were followed for an average of 67,46 ± 45,27 months after treatment (over 6.5 years). In the treated group, the mean follow-up was 48.74 ± 20.15 months (over 4 years). In the observed group the mean follow-up was longer, 96.35 ± 56.86 months (8 years). The median follow-up time in both groups was 58.88 months (range 0–316.93 months), with 54.52 months (range 0–90.13) in the treated group and 87.22 months in the observed group (25.63–316,93 months).

In the treated group of 108 patients 9.3% (10/108) died during the observation period. In the observed group of 70 patients, 7.1% (5/70) died during the observation period.

**Figure 2:**
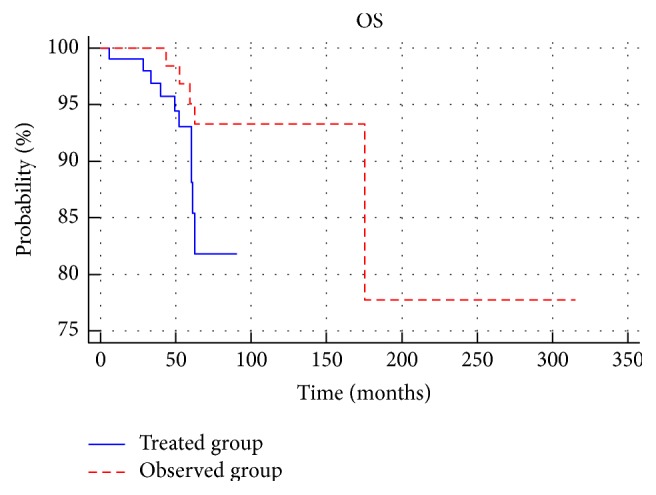
Comparison of overall survival between treated group and observed group.

**Figure 3:**
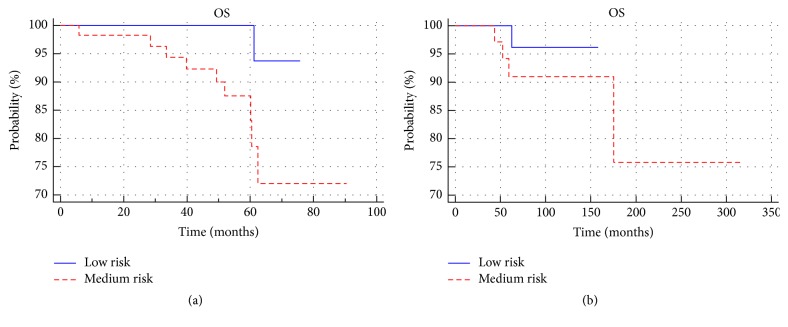
Comparison of overall survival between low- and medium-risk subgroups in the treated (a) and observed groups (b).

There were no statistically significant differences in overall survival between the treated and the observed group (*p* > 0.05) (Figure 2). In the treated group, there was a statistically significant difference in overall survival between the low- and the medium-risk patients (*p* = 0.018). There was no statistically significant difference between low risk and medium risk in the observed group (*p* > 0.05) (Figure 3). In addition, among the low-risk and medium-risk patients, no statistically significant differences in overall survival between the treated and observed group (*p* > 0.05) were reported; 3-, 5-, and 10-year OS in groups and subgroups are shown in Table 2.

### 3.2. Disease-Free Survival (DFS)

In the treated group, 4 patients (4%) were documented for recurrence during the follow-up period. The median time to recurrence was 47.54 ± 20.91 months, the median time to recurrence was 53.37 months (range 0–90 months). Two patients (2%) had a recurrence at the vaginal stump area, and in the remaining 2 patients there were distant metastases to the lung and lymph nodes of the abdominal cavity. Both local recurrences occurred among medium-risk patients. Distant metastases were documented among patients with low and medium risk. In the observed group, the mean disease-free survival was 95.4 ± 58.05 with the median 87.77 months (range 13–320 months). During the whole period of observation a recurrence of the tumor was observed in 5 patients (7%), in 4 (6%) it was a local recurrence, and in 1 patient (1%) it was lung metastases. Three patients with localized recurrence were among the low-risk patients, one among the medium-risk patients. A distant recurrence was documented in the medium-risk group.

**Figure 4:**
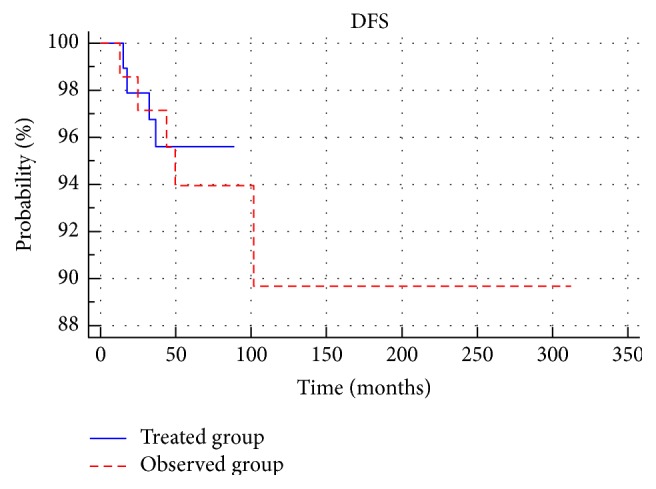
Comparison of disease-free survival between treated group and observed group.

**Figure 5:**
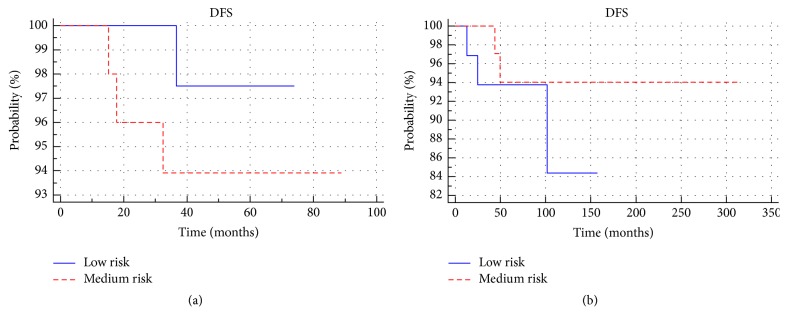
Comparison of disease-free survival between low- and medium-risk subgroups in the treated (a) and observed groups (b).

There were no statistically significant differences in disease-free survival between the treated group and the observed group (*p* > 0.05) (Figure 4). No significant difference in disease-free survival between patients with low and medium risk (*p* > 0.05) was observed in either group (Figure 5). Among the low-risk and medium-risk patients, no statistically significant differences in disease-free survival between the treated and observed group (*p* > 0.05) were reported. Three-, 5- and 10-year-old DFS in the groups and the subgroups are presented in Table 2.

### 3.3. Local Relapse-Free Survival (LRFS)

**Figure 6:**
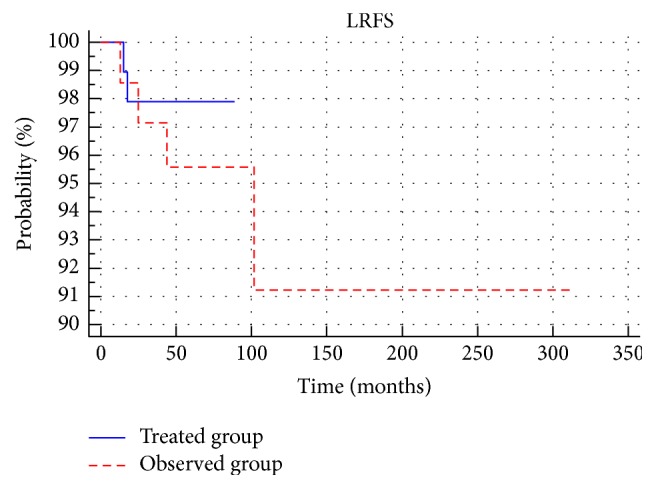
Comparison of local relapse-free survival between treated group and observed group.

**Figure 7:**
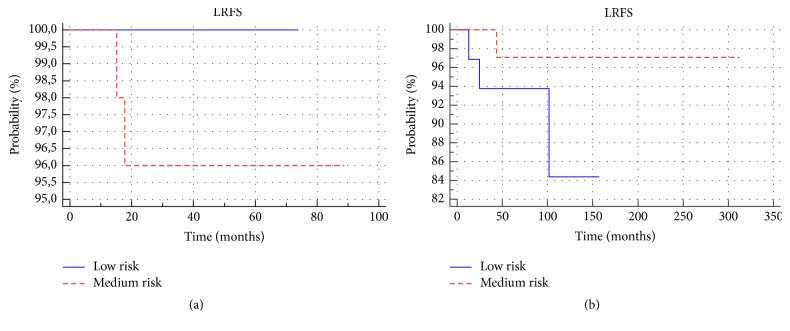
Comparison of local relapse-free survival between low- and medium-risk subgroups in the treated (a) and observed groups (b).

Local recurrences occurred in 2 patients (2%) in the treated group and 4 (6%) in the observed group (*p* > 0.05). In patients with a low risk of local recurrence none occurred in the treated group and 9% occurred in the observed group. Among the medium-risk patients, the percentage of local recurrences in both groups was similar (3%). There were no statistically significant differences in LRFS between patients in the treated group and the observed group (*p* > 0.05) (Figure 6). None of the groups showed statistically significant differences in the local relapse-free survival between low- and medium-risk patients (Figure 7). In addition, among the low-risk and medium-risk patients, no statistically significant differences in local relapse-free survival between the treated and observed group (*p* > 0.05) were reported; 3-, 5-, and 10-year-old LRFS in groups and subgroups are shown in Table 2.

### 3.4. Impact of Risk Factors

In the treated and observed groups the influence of particular factors on the prognosis was analyzed. There was a statistically significant effect of age (<70) on OS and the clinical stage on DFS. Other factors alone did not affect survival (*p* > 0.05). The results are presented in Table 3.

## 4. Discussion

Literature analysis in the low-risk group (Ia-G1, G2, and endometrioid type) indicates no benefit in overall survival from complementary brachytherapy [2]. In many cases, exclusive observation in early endometrial cancer seems to be sufficient. Very good results were obtained using also brachytherapy [12, 19, 26]. A comparison of exclusive observation and brachytherapy conducted by Sorbe et al. [9] as well as the analysis of own material indicates no statistically significant difference in the recurrence rate.

Controversy raises the value of complementary treatment in the case of FIGO IB or FIGO IA, which coincides with age-related factors such as age > 70 years, histopathological type II or mixed (according to Bokhman), low tumor descent, tumor size > 2 cm, LVSI, and histopathological grade G3. Depending on the studies in which they were evaluated and the risk factors for which they were classified in the prognostic groups, patients with recurrence risk factors were usually classified as either medium or high risk [4–6]. In the analyzed study, the medium-risk group consisted of FIGO IA patients with risk factors for recurrence (age > 70 years, histopathological type II or mixed, low tumor descent, tumor size > 2 cm, and histopathological grade G3) or patients with FIGO IB, irrespective of the presence of other factors.

Studies show that in this group of patients after the use of adjuvant brachytherapy the rate of recurrence in the vagina is very low and the rate of survival free from recurrence and overall survival is high, even when the total dose is lower than our analysis [10, 11, 13–15, 17, 18, 20–25] (Table 4). There are no large trials comparing independent brachytherapy and observation in the medium-risk group or high-intermediate-risk group. Nevertheless, PORTEC has shown that 72% of recurrences occur in the vagina, and, in GOG-99, 77.8% of all failures occurred in the vagina [4–6]. The analysis of own material in the medium-risk group indicates the lack of benefits from adjuvant brachytherapy in OS, DFS, and LRFS.

Analysis of all groups confirms the possible effect of brachytherapy on the reduction of local recurrence rate (6% in the observed group versus 2% in the treated group, NS). This is particularly evident among low-risk patients (9% in the observed group versus 0% in the treated group, NS). Unfortunately, the relapse rate in this study was too low to obtain statistical significance.

Many studies indicate deterioration of prognosis with the depth of uterine muscle invasion. Dunn et al. [27] in Cox's univariate analysis indicate that the degree of infiltration of muscle is an independent risk factor for a deterioration of the prognosis (*p* = 0.004). Some authors diminish the role of the depth of infiltration. Aristizabal et al. [28] did not show differences in 5-OS between FIGO IA and IB patients. In the analyzed group only the treated group showed a deterioration of relapse-free survival without effect on overall survival (*p* = 0.047). Among the other factors, only the elderly were affected by a deterioration of survival, which is understandable because of the likely higher incidence of coexisting diseases in this age group. The effects of advanced age on the percentage of relapses were not reported. Studies show that older age favors deterioration of overall survival by 12%, relapse-free survival by 11%, and local recurrence by 8% [27, 29]. Similarly in the study of Arenas et al. [30] age < 75 years and myometrial invasion ≤ 50% are predictors of a good outcome in endometrial cancer.

Many studies indicate an effect on prognosis of other risk factors such as tumor localization, tumor size > 2 cm, Bokhman tumor type II, or G3 grade [28, 30–33]. While the effect of G3 grade or some type of histopathologic type II tumor type does not leave room for doubt, the role of tumor size or location is questionable among many researchers [34, 35]. No statistically significant effects of the above described risk factors on OS or DFS were reported in the study group. These results may, however, be associated with a small number of patients with particular risk factors and a low number of recurrences and deaths in the analyzed group of patients.

The limitation of the study was a retrospective analysis of patients. This analysis included more than 10 years of follow-up, during which indications for adjuvant therapy were evolved to reduce brachytherapy. This causes an unequal distribution of some risk factors in both groups; however, a small percentage of them does not seem to affect the final outcomes. Another reason was the tendency to select patients depending on the type of risk factors like LUSI, size of tumor, and histopathologic type and grade.

## 5. Conclusion

Among patients with early endometrial cancer the prognosis is good, regardless of treatment administered. Brachytherapy effects on OS, DFS, and LRFS have not been demonstrated. Based on the above data, patients from the low- and intermediate-risk groups should be observed after surgical treatment. The use of brachytherapy in the low-risk group (FIGO IA, G1, 2, and no risk factors) may reduce the incidence of localized recurrences in the vagina although, due to the small percentage of relapses, these differences are not statistically significant. In the medium-risk group (IB and/or risk factors) there was no reduction in the rate of relapse after brachytherapy. Because of the lower OS in the medium-risk group, compared to the low-risk group, further research is needed to find new treatments that will improve the outcome.

The clinical stage of FIGO IB increases the risk of recurrence, although it has no effect on survival. The older age deteriorates overall survival, without affecting disease-free survival. Factors such as tumor size, low tumor location, G3, or Bokhman type II do not affect prognosis, although due to the low rate of relapses and deaths, as well as the small number of patients with particular factors, this issue requires further investigation on a larger group of patients.

## Figures and Tables

**Table 1:** Characteristics of patients.

Demographical or clinical risk factor	Treated group, *n* = 108	Observed group *n* = 70
Age of patients: median (range)	65 (47–90) years	66 (46–90) years
FIGO:		
IA	92 (85%)	59 (84%)
IB	16 (15%)	11 (16%)
Number of risk factors		
0	53 (49%)	35 (50%)
1	51 (47%)	29 (41%)
2	4 (4%)	6 (9%)
Type of risk factors		
LUSI	9 (8%)	2 (3%)
Size of tumor > 2 cm	11 (10%)	2 (3%)
Histopathologic type II	4 (4%)	13 (19%)
G3	2 (2%)	9 (9%)
Age > 70 lat	37 (34%)	23 (33%)

LUSI: lower uterine segment involvement.

**Table 2:** 3-, 5-, and 10-OS, DFS, and LRFS in treated and observed groups and among patients with low and medium risk of recurrence.

Groups and subgroups				
	3-OS	5-OS	10-OS	

Treated	96,86%,	93%,	81,8%	NS
versus				
Observed	100%	95,12%	93,3%	
Treated				
Low risk	100%	100%	93,75%,	SS *p* = 0.018
versus				
Medium risk	94,34%,	87,55%	72,01%	
Observed				
Low risk	100%	100%	96,15%,	NS
versus				
Medium risk	100%	90,95%	90,95%	

	3 - DFS	5 -DFS	10 - DFS	

Treated	96,74%,	95,58%,	95,58%,	NS
versus				
Observed	97,14%,	93,98%	89,7%.	
Treated				
Low risk	100%	97,5%	97,5%	NS
versus				
Medium risk	93,91%,	93,91%,	93,91%,	
Observed				
Low risk	93,75%	93,75%	84,38%,	NS
versus				
Medium risk	100%	94,02%,	94,02%,	

	3 - LRFS	5 -LRFS	10 - LRFS	

Treated	97,77%,	97,77%,	97,77%,	NS
versus				
Observed	97,14%,	95,57%	91,75%.	
Treated				
Low risk	100%	100%	100%	NS
versus				
Medium risk	96%	96%	96%	
Observed				
Low risk	93,75%	93,75%	84,38%,	NS
versus				
Medium risk	100%	97,06%,	97,06%,	

OS: overall survival, DFS: disease-free survival, LRFS: local relapse-free survival.

**Table 3:** 5-OS and 5-DFS in patients with risk factors.

	5-OS				5-DFS			
	Treated group	*p*	Observed group	*p*	Treated group	*p*	Observed group	*p*
FIGO IA	88,71%,	NS	96,11%,	NS	97,35%	0.047	92,92%	NS
FIGO IB	84,41%		90,00%		85,71%		100%,	
No risk factors	95,65%	0.015	100%	NS	100%	NS	100%	NS
1 risk factor	81,59%		88,14%		97,72%		92,14%	
2 risk factors	66,66%		100%		92,96%		94,28%	
Age > 70	92,86%,	0.011	95,29%,	NS	98,27%	NS *p* = 0.1	93,35%	NS
Age < 70	77,81%		94,44%		89,87%		95,23%	

**Table 4:** Fractionation schemes and survival parameters.

Author	Fractional dose	Total dose	Survival parameters	Follow-up
Sorbe et al. [9]	3–8 Gy/5 mm	18–24 Gy	LRR: 2,6%, DM: 1,3%, VR: 1,2%	-
Weiss et al. [10]	4,6–4,9 Gy/5 mm (7 Gy/surface)	13,8–14,7 Gy	Pelvic relapse 5,7%, vaginal stump relapse 1,6%, local and distance relapse 2,5%, DM: 1,6%, 5-RFS 74% in MR group and 94% in HR group	25,6 m
Chadha et al. [11]	7 Gy/5 mm	21 Gy	VR: 0%, 5-OS: 93%, 5-DFS: 87%	30 m
Anderson et al. [12]	5 Gy/5 mm	15 Gy	Pelvic relapse 3%, vaginal relapse 1%, 5-OS 84%, 5-DFS 93%,	-
Alektiar et al. [13]	6-7 Gy/5 mm	18–21 Gy	5-OS 93%, 5-DFS 97%	48 m
McCloskey et al. [14]	7 Gy/5 mm	21 Gy	Local relapse 3,4%, vaginal relapse 1,1%, vaginal and pelvic relapse 1,1%, pelvic relapse 1,1%	52 m
Ríos et al. [15]	4 Gy/5 mm	20 Gy	Vaginal stump relapse 0%, relapse in lower part of vagina 1,7%, DM 6,7%	46,7 m
PORTEC-2 [16]	7 Gy/5 mm	21 Gy	5-VR: 1,8%, 5-LRR: 5,1%, 5-OS 84,8%, 5-DFS 82,7%	45 m
Lin et al. [17]	7 Gy/5 mm	21 Gy	5-OS 86%, 5-DFS 89%	55 m
Atahan et al. [18]	5,5 Gy/5 mm	27,5 Gy	VR: 1,6%, DM 3,2%, 5-OS 96%, 5-DFS 93%	48 m
Solhjem et al. [19]	7 Gy/5 mm	21 Gy	No local relapses	23 m
Cengiz et al. [20]	7 Gy/5 mm	21 Gy	5-OS 85%, 5-DFS 92%, 5-LC 95%	54 m
Rittenberg et al. [21]	5,6 Gy/5 mm	16,8 Gy	VR: 2,3% 2-OS 97%, 5-OS 95%	32 m
Horowitz et al. [22]	7 Gy/5 mm	21 Gy	All relapse: 8,5%, vaginal relapses: 2%, 5-OS 87%, 5-DFS 90%	65 m
Rovirosa et al. [23]	5-6 Gy/5 mm	20–24 Gy	No vaginal relapses	75 m
Rovirosa et al. [23]	4–6 Gy/5 mm	24–36 Gy	No vaginal relapses	88 m
Rovirosa et al. [24]	6 Gy/5 mm	18 Gy	No vaginal relapses	41 m
Townamchai et al. [25]	4 Gy/surface	24 Gy	Vaginal relapses 1,2%, para-aortic nodes relapses 1,9%, DM 1,2%	22,8 m

LRR: locoregional relapses, DM: distal metastases, VR: vaginal recurrence, RFS: recurrence-free survival, MR: mediate risk, HR: high risk, m: months.
